# Time series forecasting for bug resolution using machine learning and deep learning models

**DOI:** 10.3389/fdata.2025.1745751

**Published:** 2025-12-19

**Authors:** Lerina Aversano, Martina Iammarino, Antonella Madau, Fabiano Pecorelli

**Affiliations:** 1 Department of Agricultural Science, Food, Natural Resources and Engineering, University of Foggia, Foggia, Italy; 2 Department of Information Science and Technology, Pegaso University, Naples, Italy; 3 Department of Engineering, University of Sannio, Benevento, Italy

**Keywords:** time series forecasting, bug resolution, machine learning and deep learning models, explainable artificial intelligence, software maintenance

## Abstract

Predicting bug fix times is a key objective for improving software maintenance and supporting planning in open source projects. In this study, we evaluate the effectiveness of different time series forecasting models applied to real-world data from multiple repositories, comparing local (one model per project) and global (a single model trained across multiple projects) approaches. We considered classical models (Naive, Linear Regression, Random Forest) and neural networks (MLP, LSTM, GRU), with global extensions including Random Forest and LSTM with project embeddings. The results highlight that, at the local level, Random Forest achieves lower errors and better classification metrics than deep learning models in several cases. However, global models show greater robustness and generalizability: in particular, the global Random Forest significantly reduces the mean error and maintains high performance in terms of accuracy and F1 score, while the global LSTM captures temporal dependencies and provides additional insights into cross-project dynamics. The explainable AI techniques adopted (permutation importance, saliency maps, and embedding analysis) allow us to interpret the main drivers of forecasts, confirming the role of process variables and temporal characteristics. Overall, the study demonstrates that an integrated approach, combining classical models and deep learning in a global perspective, offers more reliable and interpretable forecasts to support software maintenance.

## 1 Introduction

Software bugs are defects or anomalies that compromise the proper functioning of a program, causing malfunctions, unexpected behavior, or security vulnerabilities (Pressman, 2010). Their presence is inevitable in development and maintenance processes, especially in large-scale projects and open source contexts, where code complexity and the multiplicity of contributions increase the likelihood of introducing errors (Ferraro et al., 2023). The ability to quickly detect and resolve bugs is therefore crucial not only to ensure the reliability and quality of the software, but also to reduce maintenance costs and improve the end-user experience (Weiss et al., 2022; Raharja et al., 2023).

A particularly important aspect of bug management is estimating the time required to resolve them. Knowing the average resolution time in advance allows for better resource planning, optimized development team allocation, and reduced release uncertainty (Ozkan et al., 2024). In open source projects, where collaboration is often distributed and voluntary, the ability to predict resolution times becomes even more important to maintain a smooth development flow and ensure the project's long-term sustainability.

In this context, time series forecasting techniques supported by machine learning offer promising tools (Noyori et al., 2023). Predictive models not only allow for more precise estimates of future resolution times, but also for identifying recurring patterns, seasonality, and differences between projects. The introduction of deep learning methodologies allows for capturing more complex temporal dependencies, while the integration of explainable artificial intelligence (XAI) techniques promotes greater forecast transparency, making them more interpretable and reliable for developers (Kim et al., 2025; Rojat et al., 2021).

This work aims to explore these possibilities by developing a comparative approach that compares classical and deep models in local and global scenarios. From the local perspective, each project is considered as an independent series, for which dedicated models are trained; from the global perspective, on the other hand, information from multiple projects is integrated into a single model capable of learning shared patterns. Reference models such as Naive and linear regression, more advanced approaches such as Random Forest and feed-forward neural networks (MLP), and sequential models based on LSTM and GRU were examined, with global extensions that also incorporate specific embeddings for the Project variable.

The work has been developed using the public GitBugs dataset,^1^ which collects weekly time series relating to bug management processes in various open-source projects. The preprocessed and aggregated data was further enriched with process variables and temporal characteristics to build a set of features suitable for training predictive models. Starting from this dataset, the models were trained and evaluated using regression and classification metrics, providing a comprehensive assessment of their performance in terms of both numerical accuracy and discriminative ability. Finally, the resulting predictions were interpreted using explainable AI methodologies, such as permutation importance, saliency maps, and project embedding analysis, to highlight the most relevant variables and ensure transparency in the results. Thus, the paper aims not only to compare the performance of different models in forecasting bug resolution times, but also to demonstrate the value of an integrated approach that combines classical and deep learning models with explainability techniques to support more informed decisions in software maintenance.

The remainder of the paper is organized as follows. The next section presents related works, providing an overview of existing studies on bug resolution time prediction and the application of machine learning and deep learning models in this field. Section 3 details the dataset used, the preprocessing steps, the implemented models, and the evaluation protocol adopted. Subsequently, Section 4 reports the performance achieved by the local and global models, highlighting the differences between classical and deep learning approaches and discussing the findings from the explainability analyses. Finally, Section 5 summarizes the main contributions of the work, highlights its limitations, and suggests possible directions for future studies.

## 2 Related works

Research on bug prediction has developed along several lines, ranging from bug/defect prediction, to issue/bug resolution time prediction, to time-series forecasting of maintenance indicators and integration with explainable AI techniques. This work straddles these lines: it uses the public GitBugs dataset (Patil, 2025a; Patil et al., 2025), formulates the problem as a weekly time-series forecast of the average resolution time, compares classical and deep models from both a local (per-project) and global (cross-project) perspective, and provides explainability through permutation importance, saliency, and project embedding analysis.

Historically, the defect prediction literature has explored within-project and especially cross-project defect prediction (CPDP) scenarios, where knowledge is transferred between projects with different distributions (Zimmermann et al., 2009; Turhan et al., 2009). Domain adaptation techniques and more recently federated approaches have been shown to improve generalization capability (Javed et al., 2024; Wang et al., 2024), confirming the value of “global” models that learn from multiple sources and incorporate domain information to reduce distribution shift.

In parallel, the specific thread on resolution time prediction has proposed both “classic” approaches–from single-bug fast/slow classification (Bhattacharya and Neamtiu, 2011; Zhang and Kim, 2013) to analyses on industrial data–as well as recent approaches that integrate text, metadata, and process characteristics. The recurring evidence is that combining structured signals (priority, backlog, team load, project dynamics) with temporal features yields more robust estimates than using text alone. Recent interest also includes LLM-based models to quickly distinguish long from short fix times (Nastos and Chatzigeorgiou, 2025a), useful for triage, but not always accompanied by evaluations over multi-step or multi-project time horizons. Furthermore, studies aimed at explainability confirm the importance of providing transparency in resolution time estimates (Nastos and Chatzigeorgiou, 2025b; De Luca et al., 2023).

On the methodological side, deep learning surveys for time series offer a framework for choosing between classical models and sequential architectures (RNN/LSTM/GRU, Transformers), highlighting open questions on generalization, temporal validation, and explainability. The adoption of time series-specific XAI tools (e.g., saliency maps, perturbation/permutation) is considered crucial to make the models usable in real-world contexts (Liu et al., 2024; Lim and Zohren, 2021; Rojat et al., 2021). Our joint use of permutation importance (for tree models) and gradient-based saliency (for sequential models) follows these recommendations.

Given these methodological considerations, it is essential to have large and diverse datasets that allow us to compare classical and deep approaches in realistic scenarios. This is where the use of GitBugs comes in. This public dataset enables various lines of analysis on bug reports and allows us to systematically test the effectiveness of local and global models.

The GitBugs dataset was introduced to enable various bug report analysis tasks–duplicate detection, triage, RAG, resolution prediction, and temporal analysis (Patil, 2025a,b; Patil et al., 2025). Work related to GitBugs has primarily focused on textual tasks (similarity, duplicates, triage) and the evaluation of NLP/embedding models; the “resolution prediction” component is mentioned as a use case enabled by the dataset, but to date there has been no systematic comparison between classical and deep models from a time-series perspective, nor a local vs. global evaluation of the weekly forecast of the average resolution time. This is where our contribution lies.

Unlike per-issue or class-based fix time studies, we explicitly model a weekly time series of the average resolution time per project, thus capturing seasonality and dependencies in the maintenance process rather than limiting ourselves to a binary classification. In line with CPDP evidence, we propose and evaluate global models (Random Forest with project one-hots and LSTM with project embeddings) that learn shared cross-project patterns, comparing them with their local counterparts. This type of systematic comparison on the forecast bug-resolution time at the multi-project level is rare in existing work. Finally, we provide explainability consistent with the nature of the models: permutation importance for tree ensembles and saliency/embedding inspection for sequential models, following the XAI time series guidelines (Ozkan and Tosun, 2024).

## 3 Methodology

The methodology adopted in this work was designed to systematically address the problem of forecasting mean time to resolve bugs, while ensuring transparency and reproducibility. The section is divided into five main subsections. The Dataset section describes the data corpus used, with particular reference to the GitBugs dataset and its characteristics. The Preprocessing Step subsection illustrates the data cleaning, transformation, and aggregation operations required to convert the raw reports into weekly time series suitable for forecasting. The Models section presents the various algorithms adopted, including both classical models and deep learning architectures, in local and global configurations. The Validation subsection describes the metrics, evaluation procedures, and comparison criteria used to ensure a systematic performance analysis. Finally, the Explainability section introduces the interpretability techniques applied to the models, with the aim of making decisions more transparent and providing useful insights into the role of variables and the learned temporal dynamics.

### 3.1 Dataset

For the experimental evaluation, we used the GitBugs dataset (Patil, 2025a; **?**,b; Patil et al., 2025), a public resource that collects bug reports from various open-source projects hosted on GitHub. The dataset was designed to enable various analysis tasks, including duplicate detection, triage, retrieval augmented generation (RAG), resolution time prediction, and temporal analysis. The corpus contains bug reports extracted from numerous popular software systems, including Apache Spark, Kubernetes, Cassandra, Firefox, Hadoop, HBase, Thunderbird, Mozilla Core, Seamonkey, and VSCode.

Table 1 shows the distribution of bug reports across projects included in GitBugs. Significant heterogeneity is observed in terms of dataset size: projects like Mozilla Core and VS Code have tens of thousands of reports, while smaller systems like Seamonkey or Hadoop have small volumes. This variability makes the dataset particularly interesting for comparative analyses, as it allows us to evaluate the performance of models both in contexts with a high density of reports and in rarer, sparsely populated scenarios. Furthermore, the presence of different domains (distributed databases, big data frameworks, browsers, IDEs, email clients) provides a variety that allows us to test the generalization ability of forecasting models.

**Table 1:** Distribution of bug reports in the GitBugs dataset by project.

**Project**	**Totale bug report**
Cassandra	4,612
Firefox	28,824
Hadoop	2,503
HBase	5,403
VS Code	32,829
Spark	20,275
Thunderbird	15,192
Mozilla Core	85,673
Seamonkey	1,076

Each bug report in the dataset is described by a rich set of features. In addition to textual attributes (title, description, comments), metadata related to bug management is available, including:

- Identifier of the bug and the source project.
- Timestamps of the bug's creation and closure, which allow us to calculate the resolution time.
- Associated labels and categories (priority, severity, type).
- Information about the developer or team involved in the resolution.
- Bug status (open, closed, in progress).

To better contextualize the data characteristics and the complexity of the forecasting task, a brief exploratory analysis of the weekly time series was also conducted. This analysis highlights some recurring properties across the different projects, useful for understanding the behavior of resolution time and justifying the methodological choices adopted in the following sections. A preliminary inspection of the weekly time series of the projects included in the dataset reveals strong heterogeneity in the evolutionary dynamics of resolution time. Some projects exhibit clearer trends (increasing or decreasing), others exhibit regular fluctuations due to seasonal components, while in several cases, irregular fluctuations emerge associated with sudden increases in the backlog or changes in the flow of reports. The combination of high-frequency noise, medium-term trends, and possible cyclicalities makes the forecasting problem particularly challenging, justifying the adoption of dedicated temporal transformations and models capable of capturing nonlinear dynamics and long-term dependencies.

### 3.2 Preprocessing step

The goal of preprocessing was to transform the raw bug reports from the dataset into weekly time series suitable for forecasting the average resolution time. First, the temporal information was normalized and aligned: the creation and closure dates of bugs, sourced from heterogeneous formats and trackers, were made consistent into a single time reference. From this, the resolution time was calculated for each resolved issue; unresolved cases were excluded from the duration statistics.

The data was then aggregated on a weekly basis (consistent and non-overlapping weeks), producing summary indicators of the maintenance process for each week. Specifically, the target series was the average weekly resolution time (along with the median and 90th percentile to capture the tail of the distribution), while the exogenous signals were the open and closed bug counts per week, with priority normalization into three levels (high, medium, low) and the corresponding percentage shares. To represent load dynamics over time, a cumulative backlog was also constructed, obtained by accumulating the difference between weekly openings and closings.

To facilitate the learning of seasonal patterns and process inertia, the series was enriched with temporal characteristics (year, ISO week, month, seasonal sinusoidal components) and with lagged versions of the target variable (1, 2, and 4 weeks) as well as rolling statistics (mean and standard deviation) computed over 4-, 8-, and 12-week windows. These transformations, applied in a causal manner (using only past information), do not alter the observed data, but provide the model with synthetic signals about persistence, fluctuations, and seasonality of the resolution time.

The entire procedure was applied consistently to each project, producing homogeneous weekly series that were then consolidated into a single multi-project dataset. This framework allows for both local (one model per project) and global (a single model that learns regularities shared across projects) analyses, facilitating systematic comparisons between model families and generalization scenarios.

During the pre-processing phase, all bug reports without a closed date were excluded, as calculating resolution times necessarily requires both the creation and closing dates. In the GitBugs dataset, this resulted in the elimination of a significant number of unresolved reports, with considerable variability across projects depending on activity intensity, maintenance policies, and backlog size. This removal resulted in an overall reduction of approximately 3.6% in reports usable for calculating resolution times. Excluding these reports can introduce biases in the construction of the time series. In particular, bugs that remain open for long periods often tend to be more complex or receive a lower priority; therefore, their removal can lead to an underestimation of the average weekly resolution time. Similarly, periods characterized by slowdowns or backlog accumulation can be mitigated, as bugs opened during these phases do not contribute to the time statistics. To mitigate these effects, operational indicators such as weekly open and closed bugs and cumulative backlog were retained in the dataset, which still allows us to indirectly capture maintenance process pressures even in the presence of unresolved issues.

### 3.3 Temporal feature engineering

To explicitly model the temporal dimension of the bug fixing process, the weekly series was enriched with a set of engineered features, designed to provide the model with memory, trend, and seasonality signals. All transformations were computed in *causal* mode, that is, using only the information available up to week *t*, avoiding any form of leakage.

Lag features: let *y*_*t*_ be the average resolution time observed at week *t*. To capture the short-term inertia effect, lagged versions of the target variable were introduced:


$$y_{t-1},\;y_{t-2},\;y_{t-4}.$$

These features allow models to exploit the persistence of the maintenance process, where delays or accelerations tend to propagate over the following weeks.

Rolling statistics: to represent the trend and stability over longer time horizons, the moving average and moving standard deviation of the target variable were calculated:


$$\begin{array}{cc} {\text{rollmean}}_{w}\left(t\right)= & \frac{1}{w}\overset{w-1}{\underset{i=0}{\sum }}y_{t-i},\\ {\text{rollstd}}_{w}\left(t\right)= & \sqrt{\frac{1}{w}\overset{w-1}{\underset{i=0}{\sum }}{\left(y_{t-i}-{\text{rollmean}}_{w}\left(t\right)\right)}^{2}} \end{array}$$

with *w*∈{4, 8, 12} week windows. Moving averages summarize recent trends, while moving standard deviations highlight periods of greater or lesser variability in the maintenance process.

Seasonal signals: because software maintenance processes can exhibit annual cyclicity, the ISO week of the year has been encoded using sinusoidal components:


$$\begin{array}{cc} \text{week\_sin}\left(t\right)= & \sin\left(\frac{2\pi \cdot \text{week}\left(t\right)}{52}\right),\\ \text{week\_cos}\left(t\right)= & \cos\left(\frac{2\pi \cdot \text{week}\left(t\right)}{52}\right). \end{array}$$

This continuous representation eliminates the discontinuity between week 52 and week 1, allowing the models to learn seasonal patterns more seamlessly.

Overall, these temporal transformations complement the original weekly indicators by providing synthetic signals about the persistence, medium-term fluctuations, and seasonal cyclicality of bug fix time. In combination, they strengthen the ability of both classical models and sequential neural networks to capture the typical dynamics of the maintenance process.

### 3.4 Models

This study compared models from three main families: (i) a naïve baseline model, (ii) classical regression and ensemble models, and (iii) sequential deep learning models, in local (per-project) and global (cross-project) configurations. Below, we briefly describe each class, the rationale behind their choice, and the literature surrounding them.

#### 3.4.1 Naïve baseline

The naïve model, frequently used as a benchmark in forecasting studies, simply assumes that the future prediction coincides with the last observed value of the target (persistence). It is not sophisticated, but serves as a benchmark to assess whether more complex methods add real value, as is commonly the case in forecasting competitions (M-Competitions highlight that complex methods do not always win over simple baselines) (Makridakis et al., 2020).

#### 3.4.2 Classical models: linear regression and random forest

Among traditional methods, we included linear regression to estimate linear relationships between features and mean resolution time. However, the complex and nonlinear nature of the software maintenance process suggests that more flexible models may be more effective. For this reason, our primary approach in the classical class is Random Forest Regressor. Tree ensembles (forests) are widely used in software analytics scenarios (bug prediction, defect fix time) due to their ability to capture nonlinear relationships without requiring intensive preprocessing and their interpretability (feature importance) through techniques such as permutation importance. In comparative studies between ML and DL models, Random Forest is often a benchmark competitor and in many cases provides robust performance (although it does not always dominate in the presence of deep temporal dependencies) (Ghotra et al., 2015).

#### 3.4.3 Sequential deep learning models: LSTM

To capture the temporal dependencies (inertia, cyclicity, long-term memory) implicit in the bug-fixing process, we adopted models based on Long Short-Term Memory (LSTM), a variant of RNN particularly suited for sequences with extensive dependencies (Hochreiter and Schmidhuber, 1997). LSTMs are widely used in real-world time series forecasting because they mitigate the vanishing gradient problem and allow learning longitudinal patterns. In distributed forecasting contexts across multiple series, some recent proposals suggest training “global” LSTM networks that learn from multiple series simultaneously, capturing patterns shared across projects (Bandara et al., 2020). In our global setting, the LSTM network is enriched with project embeddings (i.e., a numerical representation of the project itself) that allow the model to adapt the forecast to the specific context of the project, while still benefiting from experience learned from other projects. This strategy is similar to cross-series techniques that seek to exploit similarity between series to improve generalization (e.g., clustering of series by groups) (Montero-Manso et al., 2020).

### 3.5 Experimental setup

All experiments were conducted using a consistent and reproducible configuration, without resorting to systematic hyperparameter tuning procedures. This choice was driven by the desire to ensure a fair comparison between local and global models, prioritizing stability and comparability over excessive performance optimization. The hyperparameters were therefore set to standard values commonly used in the literature on time series forecasting and software maintenance process analysis. For the classical models, linear regression was used in its conventional form, while the naïve model adopted the persistence approach, assuming that the future value coincides with the last observed value. The Random Forest, which represents the primary nonlinear baseline, was configured with a large number of trees (400 in the local configuration and 600 in the global one), keeping all other parameters at the default values provided by scikit-learn. This choice is consistent with numerous works in the fields of defect prediction and resolution time modeling, where this configuration has proven robust even in the absence of hyperparameter optimization. For deep learning models, an LSTM architecture was adopted, designed to capture the sequential structure of the data, employing a 12-week time window and a 64-unit recurrent layer, followed by a 32-unit dense intermediate layer. Training was performed using the Adam optimizer with a learning rate of 10^−3^, using a batch of 32 elements and a maximum of 100 epochs, with an early stopping mechanism after ten epochs without improvement. In the global setup, project information was incorporated via a dimension-eight embedding, concatenated to the time sequence to allow the model to learn patterns shared across projects while maintaining local specificities. The validation phase followed a strictly temporal strategy. For each project, the data set was divided into three consecutive segments: an initial portion used for training, a subsequent twenty-four-week window for validation, and, finally, the final twenty-four weeks for testing. In the overall configuration, these segments were constructed separately for each project and subsequently concatenated, ensuring temporal causality and the ability to compare performance both at the individual and aggregate levels.

### 3.6 Validation

Model evaluation was conducted using a time-based framework that respects the chronological order of observations, thus avoiding any form of information leakage. Three periods were distinguished for each series: a training window, a validation window, and a test window, corresponding to the first few historical weeks, the intermediate weeks, and the final weeks, respectively. Validation was used to select parameters and identify the best model, while the final test provides an unbiased estimate of predictive performance. To ensure a realistic forecasting setup and prevent any form of data leakage, each project in the dataset was temporally divided into three contiguous blocks: training, validation, and testing. The division was performed separately for each project, strictly maintaining the chronological order of the weeks.

To clarify the strategy adopted in temporally splitting the data, Table 2 shows an example of the chronological partition applied to three representative projects. For each project, all weekly observations were divided into three contiguous segments: an initial training period used to train the models, a 24-week validation block used for hyperparameter selection and early stopping activation, and a final 24-week testing block intended exclusively for evaluation on unseen future data. This strategy rigorously maintains the temporal order of events and avoids any form of data leakage, ensuring that the evaluation reflects a realistic forecasting scenario.

**Table 2:** Example of chronological train–validation–test split for three projects.

**Project**	**Training**	**Validation**	**Test**
Thunderbird	Week 1–180	Weeks 181–204	Weeks 205–228
VS Code	Week 1–230	Weeks 231–254	Weeks 255–278
Jenkins	Week 1–160	Weeks 161–184	Weeks 185–208

Performance was measured using regression metrics widely used in forecasting: Mean Absolute Error (MAE), which quantifies the average error in original units (hours); Root Mean Squared Error (RMSE), which penalizes large errors more; and Mean Absolute Percentage Error (MAPE), which expresses the average relative deviation as a percentage, facilitating comparison between projects of different scales.

Alongside the regression analysis, a binary classification analysis was also conducted, in which the mean resolution time values were divided into “high” and “low” according to data-driven thresholds (median or quantile). In this scenario, the models were evaluated in terms of accuracy, precision, recall, and F1 score, to capture their ability to distinguish more or less costly maintenance situations.

This dual approach (regression and classification) allows us to assess the models' performance both in providing accurate quantitative estimates and in supporting decision-making scenarios in which it is important to distinguish critical projects or periods from more manageable ones.

### 3.7 Explainability

A crucial aspect of this study concerns the ability to make predictive models interpretable, in line with the recommendations of recent literature on Explainable AI applied to time series (Rojat et al., 2021; Ozkan and Tosun, 2024). Although accuracy metrics provide a synthetic measure of performance, they are not sufficient to understand the reasons for predictions or to support reliable decisions in software maintenance processes.

For Random Forest-based models, Permutation Feature Importance was applied, which evaluates the impact of each variable on the predictive result by measuring the increase in error when the feature values are randomly permuted. This robust and intuitive technique allows us to identify the variables that most influence the average resolution time, such as the weekly backlog or the proportion of high-priority bugs.

In the case of LSTM neural networks, interpretability was addressed using gradient-based methods, specifically saliency maps computed with respect to the input sequence. These maps highlight the weeks and exogenous variables most relevant to the forecast, allowing us to verify whether the model truly exploits the expected temporal patterns (seasonality, inertia, local anomalies). Furthermore, in the global model enriched with project embeddings, the vector representation learned for each project was analyzed to explore similarities and differences between projects in terms of maintenance behavior.

This dual approach to explainability, tailored to different types of models, not only increases forecast transparency but also provides useful insights for managing maintenance processes: for example, identifying the most critical variables or understanding how different projects share (or do not) common temporal patterns.

For the global LSTM model, saliency estimation was obtained via first-order gradient saliency, directly calculating the gradient of the prediction with respect to the input features. To this end, TensorFlow's self-differentiation mechanism was used with a single pass of tf.GradientTape(). The final saliency associated with each feature was calculated as the average of the absolute value of the gradients over a random sample of 5,000 sequences drawn from the validation and test set (or over the entire set when the number of available sequences was smaller). This configuration allows for a robust estimate of the local importance of the features while maintaining a low computational cost, even for the global model that aggregates heterogeneous sequences from different projects.

## 4 Results

This section reports the experimental results obtained for both local and global models, along with their explainability.

### 4.1 Local forecast

In the local configuration, each model was trained exclusively on data from a single project, reflecting the scenario in which teams develop customized solutions without cross-project collaboration. The average performance reported in Table 3 clearly shows the limitations and strengths of the different model families.

**Table 3:** Average performance of models in local configuration per project.

**Modello**	**MAE (h)**	**RMSE (h)**	**MAPE (%)**	**Accuracy**	**Precision**	**F1**
Naïve	2,381.7	3,181.3	406.7	0.65	0.67	0.75
Random Forest	**676.2**	**1,024.4**	**36.4**	**0.92**	**0.93**	**0.94**
LSTM	3,128.4	4,059.7	210.9	0.31	0.21	0.22

The best F-score results per project are given in bold.

The naïve baseline, despite being conceptually simple (it uses the last week's value as a future prediction), paints an interesting picture. From the classification perspective, it achieves apparently acceptable values (Accuracy 0.65, F1 0.75), but the regression metrics are extremely high (MAE over 2,300 h, MAPE over 400%). This indicates that the naïve model tends to predict well only when the series is stationary or in the absence of sudden variations, but fails completely in the presence of large fluctuations, thus offering the illusion of good performance when evaluated with non-robust metrics. The Random Forest emerges as the most effective model at the local level. With an average MAE of less than 700 h and a low MAPE of 36%, RF demonstrates a high ability to capture nonlinear relationships between backlog, priorities, and temporal dynamics. From a classification perspective, the Accuracy (0.92) and F1 (0.94) values also confirm the model's stability and generalization ability. In contrast, LSTM networks do not show satisfactory performance in the local context: regression errors are very high (MAE about 3,128 h, MAPE about 211%) and classification metrics are particularly low (Accuracy 0.31, F1 0.22). This result reflects the difficulty of sequential architectures in learning robust patterns from relatively short and noisy time series, such as those from a single project. In practice, LSTM fails to generalize in the absence of sufficiently large and varied data, suffering from overfitting or underutilization of its modeling capacity.

A qualitative analysis of individual projects also suggests that naïve models perform better in cases with high temporal stability, while Random Forests adapt well even to projects characterized by strong variability. LSTMs, while potentially powerful, require pooling of multiple series (global scenario) to exploit shared patterns. Overall, the local results demonstrate that, although simple baselines and classical models provide robust performance in individual contexts, deep learning models require multi-project approaches to fully realize their potential. This finding directly motivates the global analysis.

A more detailed analysis of the results by project, reported in Table 4, confirms the picture emerging from the average performance, but also highlights significant differences between the various systems. For some projects with relatively stable data sets, such as Firefox, Thunderbird, and Spark, the naïve model already achieves good results, with MAPE values below 60% and high classification metrics. In these cases, the temporal persistence of the solution process makes the simple projection of the last value a surprisingly competitive approach. However, Random Forest still manages to further improve accuracy, reducing the mean error (MAE) to around 200 h in Spark and less than 400 h in Thunderbird, achieving near-perfect performance even in classification metrics (Accuracy and F1 equal to 1.00 in more than one case). In the Cassandra, Hadoop, and Hbase projects, which feature noisier and more irregular data sets, the differences between the models become more pronounced. Here, the naïve model shows clear limitations, with very high MAPE values (up to over 700%), while the Random Forest proves robust, maintaining lower errors (MAE in the range of 400–2,000 h depending on the project). LSTMs, on the other hand, fail to adapt adequately: in almost all cases, they report zero classification metrics and very high error values, a sign of an intrinsic difficulty in learning useful patterns from short, highly variable data sets when working at the single-project level. Finally, VS Code represents an intermediate case: the naïve model achieves reasonable performance (MAE about 1,371 h, Accuracy = 0.75), but the Random Forest manages to reduce the average error by more than half while maintaining perfect classification. Here too, the LSTMs show no improvement, with errors exceeding 3,800 h. Overall, the project-specific results reinforce the evidence that Random Forests are the most reliable model in the local setting, adapting well to both more stable and more variable contexts. Naïve baselines remain useful only in scenarios with strong temporal inertia, while sequential deep learning models systematically suffer from data scarcity and fail to exploit their potential in the absence of multi-project pooling.

**Table 4:** Model performance in the local configuration (test set) per project.

**Project**	**Model**	**MAE**	**RMSE**	**MAPE%**	**Accuracy**	**Precision**	**F1**
Cassandra	LSTM	2,149.24	3,300.84	658.81	0.25	–	–
Cassandra	Naïve	1,826.94	2,740.22	1,202.09	0.46	0.62	0.61
Cassandra	RF	401.52	778.00	66.92	0.92	0.94	**0.94**
Firefox	LSTM	4,591.36	4,880.46	83.93	–	–	–
Firefox	Naïve	1,484.56	2,137.48	31.94	0.92	0.96	0.96
Firefox	RF	526.92	873.07	9.59	0.99	0.99	**0.99**
Hadoop	LSTM	3,474.00	5,418.91	468.82	0.33	–	–
Hadoop	Naïve	4,602.40	6,247.83	831.73	0.58	0.67	0.71
Hadoop	RF	955.85	1574.08	40.80	0.96	0.94	**0.97**
Hbase	LSTM	5,174.00	9,403.84	71.15	0.33	–	–
Hbase	Naïve	5,792.78	9,513.61	743.87	0.42	0.59	0.59
Hbase	RF	2,010.98	4,839.73	66.44	0.83	0.81	**0.89**
Spark	LSTM	389.59	562.05	59.39	0.58	0.55	0.71
Spark	Naïve	430.62	588.49	57.88	0.58	0.64	0.59
Spark	RF	183.04	236.50	25.92	0.92	0.94	**0.93**
Thunderbird	LSTM	2,930.18	3,501.88	103.68	0.33	–	–
Thunderbird	Naïve	612.62	866.31	20.09	0.92	0.96	0.94
Thunderbird	RF	399.19	605.92	13.57	0.99	0.99	**0.99**
VS code	LSTM	3,829.85	4,507.23	112.67	0.33	0.25	0.29
VS code	Naïve	1,371.28	1,712.04	44.69	0.75	0.75	0.75
VS code	RF	593.70	769.06	18.55	0.99	0.99	**0.99**

The best F-score results per project are given in bold.

### 4.2 Global forecast

In the global configuration, a single model was trained by jointly exploiting all the projects' time series, including the project encoding as a feature (one-hot for Random Forest, embedding for LSTM). It is important to note that, in the global configuration, the naïve baseline was not reported. This is because the naïve model, by its nature, simply predicts that the next value coincides with the last observed value in the single time series. This approach makes sense in the local configuration, where comparison with more complex models allows us to assess whether they exceed simple persistence. However, in the global configuration, the naïve model could not exploit any cross-project information, effectively reducing itself to an independent local prediction for each project. For this reason, the global results only report models capable of learning shared regularities across projects, such as Random Forest and LSTM.

The average results on the test set show a clear difference between the two families of models as reported in Table 5.

**Table 5:** Average performance of models in the global configuration (Test set).

**Model**	**MAE**	**RMSE**	**MAPE%**	**Accuracy**	**Precision**	**F1**
RF_Global	612.12	1169.75	55.45	0.91	0.91	**0.93**
LSTM_Global	2,377.11	3,466.23	536.26	0.73	0.73	0.82

The best F-score results per project are given in bold.

The global Random Forest model achieves significantly better performance, with an average MAE of approximately 612 h and a MAPE% around 55%, while maintaining high classification accuracy (0.91) and an F1 score above 0.93. This suggests that, despite their simplicity, tree-based ensembles can robustly capture cross-project regularities. In contrast, the global LSTM model, while theoretically benefiting from the ability to model long-term temporal dependencies, exhibits very high errors (MAE about 2,377 h, MAPE% over 500%), with lower classification performance (Accuracy 0.73, F1 0.82). This indicates that, at least in our setting and with the size of the available dataset, sequential architectures failed to generalize effectively in predicting solution time.

Overall, the results confirm the effectiveness of global Random Forest-based models for cross-project mean-to-solve time forecasting, while highlighting critical issues in the direct use of sequential deep learning models without further optimizations or regularization strategies.

#### 4.2.1 Explainability results

**Figure 1:**
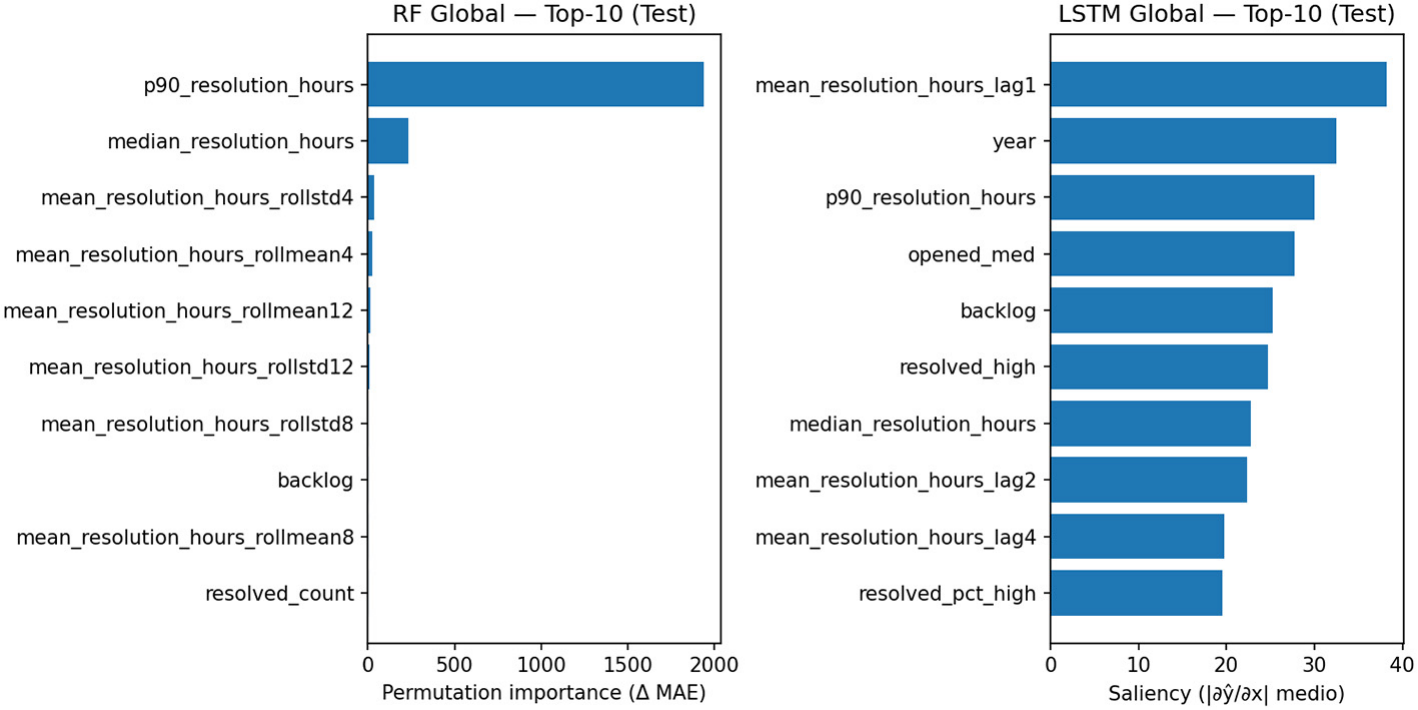
Top-10 salient features for the global LSTM model on the test set.

To interpret the global models, we adopted explainability techniques consistent with their nature: Permutation Importance for the Random Forest (measures the change in error Δ(*MAE*) when a feature is permuted) and gradient-based saliency map for the LSTM (estimates the average influence |δ(ŷ)/δ(*x*)| of the features along the sequence). The Summary Figure 1 (RF vs. LSTM) and Tables 6, 7 report the top 10 attributes in the Test Set.

**Table 6:** Top-10 feature per random forest global (permutation importance, test).

**Rank**	**Feature**	**Permutation importance (ΔMAE)**
1	p90_resolution_hours	1,940.934
2	Median_resolution_hours	231.845
3	Mean_resolution_hours_rollstd4	35.192
4	Mean_resolution_hours_rollmean4	22.231
5	Mean_resolution_hours_rollmean12	12.646
6	Mean_resolution_hours_rollstd12	7.082
7	Mean_resolution_hours_rollstd8	5.536
8	Backlog	5.256
9	Mean_resolution_hours_rollmean8	4.833
10	Resolved_count	3.276

**Table 7:** Top-10 feature per LSTM global (gradient-based saliency, test).

**Rank**	**Feature**	**Saliency (|*∂ŷ*/*∂x*| medio)**
1	Mean_resolution_hours_lag1	38.202
2	Year	32.542
3	p90_resolution_hours	30.065
4	Opened_med	27.717
5	Backlog	25.263
6	Resolved_high	24.774
7	Median_resolution_hours	22.815
8	Mean_resolution_hours_lag2	22.338
9	Mean_resolution_hours_lag4	19.778
10	Resolved_pct_high	19.595

For the global Random Forest, a clear dominance of robust statistics of the resolution time distribution emerges: the 90th percentile (p90_resolution_hours) is by far the most important predictor, followed by the median. Aggregate target memories (rolling mean/std over 4–12 week windows) and a workload signal (backlog) complete the analysis. This pattern indicates that the set of trees primarily leverages stable tail and historical variability indicators to predict the weekly mean, proving reliable in cross-project contexts.

The global LSTM, on the other hand, builds the forecast by combining explicit target memories (lag1/lag2/lag4) with calendar cues (year), distribution tails (p90), and weekly workload (opened_med, backlog, resolved_high, resolved_pct_high). This profile suggests that the neural network jointly leverages temporal inertia and operational cues; however, the greater dispersion of importance across multiple features is consistent with the performance variability observed in the global results.

In summary, explainability confirms two complementary strategies: (i) the global RF favors a few robust and stable predictors (distribution and aggregate memories), with a more “rigid” but highly effective behavior; (ii) the global LSTM integrates short-term memory and exogenous cues, showing sensitivity to more factors in the maintenance process, but with more variable performance. This evidence is consistent with the quantitative results: in our setting, global RF achieves the best balance between accuracy and stability, while LSTM benefits from multi-project training but does not always generalize uniformly across systems.

## 5 Conclusions

This work addressed the problem of forecasting bug resolution times in open-source projects by comparing classical machine learning models and sequential deep learning models, both in local (single-project) and global (cross-project) configurations. Using the GitBugs dataset, raw bug reports were transformed into homogeneous weekly time series and enriched with temporal and process characteristics, allowing for a systematic evaluation of the prediction models. The results showed that, at the local level, Random Forest consistently outperforms both the naïve baseline and deep sequential models, ensuring robust performance even on projects with marked variability. However, the global analysis highlights the additional benefits of cross-project learning: the global Random Forest offers the best tradeoff between accuracy and stability, while the global LSTM, while less competitive in terms of mean error, captures complementary temporal dynamics and derives additional value from explainability analyses. The integration of explainable AI techniques further strengthened the contribution of this work. Importance analysis using permutation importance confirmed the central role of distributional statistics and backlog dynamics in tree models, while saliency maps for the LSTM highlighted a greater dependence on time lags, load indicators, and seasonal signals. This dual interpretative approach not only increases model transparency but also provides useful insights for managing maintenance processes.

One aspect that clearly emerged concerns the limited performance of the LSTM model, especially in the local configuration. This finding is consistent with what is reported in the literature on time series applied to software engineering: LSTMs require large amounts of homogeneous sequential data to effectively learn stable temporal dependencies. In our weekly series, each project has a relatively small number of observations and exhibits irregular maintenance dynamics, characterized by sudden fluctuations in bug openings and closures. This combination makes it difficult for the network to fully exploit its long-term memory and promotes unstable behavior, with a high risk of overfitting. Furthermore, weekly granularity reduces the temporal richness of the signal: short-term patterns–intraweekly fluctuations, bursts of activity, sudden buildups of backlog–are compressed into aggregate indicators, resulting in the LSTM receiving an already “smoothed” and less informative input signal compared to scenarios with daily or issue-level data. Rolling statistics and lags help compensate for this loss of detail, but they are not sufficient to make the sequence fully descriptive. Furthermore, in local models, the network cannot benefit from the experience of multiple projects: each series, alone, does not offer enough heterogeneity to guide the learning of shared patterns. This explains why the global LSTM, while not outperforming the Random Forest, performs better than its local version. Looking ahead, these limitations open up interesting directions for future work. The adoption of attention-based or Transformer-based architectures for time series could reduce the dependence on sequence length, favoring the dynamic selection of the most relevant information. Multi-resolution approaches, combining daily, weekly, and longer-term signals, would allow for the simultaneous capture of rapid changes and underlying trends. Finally, data augmentation techniques for time series, hybrid tabular-sequential models, and cross-project transfer learning strategies could further strengthen the model's ability to generalize even to relatively small datasets, typical of the software maintenance domain.

Further evidence emerging from the results concerns the robustness of the Random Forest in the global configuration. The heterogeneity of patterns observed across projects–irregular backlog fluctuations, variations in weekly openings and closings, differences in priorities and distributions of fix times–translates into an advantage for tree ensembles. The forest, in fact, builds each tree on different subsets of data and features: the greater the variety of configurations observed during training, the greater the structural diversification of the trees and the more stable the aggregate prediction. In this sense, multi-project data functions as a natural form of data augmentation, reducing variance and increasing the model's ability to generalize even to dynamics not perfectly observed in the single project. This explains why the global Random Forest maintains robust and consistent behavior, outperforming both the corresponding local version and the LSTM model, which is more sensitive to the limited length of the series and the lack of temporal regularity.

Some limitations remain to be considered. First, the dataset, although large and diverse, is limited to open-source projects and may not fully reflect the dynamics of industrial contexts. Second, sequential models suffered from data scarcity, suggesting that larger datasets or advanced architectures (such as Transformers or hybrid ensembles) could lead to significant improvements. Finally, the analysis was conducted on weekly aggregated series: extensions to the daily or issue-level could further enrich the perspective.

The implications that have emerged highlight how global models represent a promising direction for forecasting in software maintenance, as they exploit shared regularities across projects and increase robustness compared to purely local models. Furthermore, the integration of explainability tools ensures that forecasts are not only accurate but also interpretable, a key requirement for practical adoption in real-world scenarios.

Another important aspect concerns the computational costs associated with the different models. In our experiments, the local approach has modest training times: both the naïve baseline and the classical models (regression and Random Forest) train quickly on each project, thanks to the small size of the time series. However, complexity increases when switching to the global configuration, where all the time series are concatenated or processed jointly. In this scenario, the global Random Forest remains efficient, with training times on the order of a few minutes, while deep learning models (global LSTM) require significantly longer training times, due both to sequence generation and the greater number of parameters to optimize. In professional contexts, the difference between local and global models therefore translates into a practical trade-off: global models require longer training times, but offer more stable behavior and greater generalization ability; local models are lighter and easily retrained, but are more sensitive to the variability of the individual project. These considerations are useful for those who intend to integrate bug-resolution time forecasting into continuous maintenance pipelines, where the balance between predictive accuracy and computational sustainability becomes crucial.

For future research, we intend to explore hybrid strategies that combine tree ensembles and sequential models, as well as extensions toward multimodal forecasting that integrates textual and process metadata. Another line of research concerns federated or privacy-preserving approaches, which allow predictive knowledge to be shared between organizations without exposing sensitive data.

## Data Availability

The original contributions presented in the study are included in the article/supplementary material, further inquiries can be directed to the corresponding author.

## Footnotes

1
https://github.com/av9ash/gitbugs
